# Supplementary material for: Heart rate variability before and after 14 weeks of training in Thoroughbred horses and Standardbred trotters with different training experience
Source: PLoS One. 2021 Dec 9;16(12):e0259933. doi: 10.1371/journal.pone.0259933 (PMC8659354; doi:10.1371/journal.pone.0259933)
Supplement: Attachment — Submitted filename: Review PONE-D-21-19666.docx [file pone.0259933.s001.docx]

**Review of the manuscript No: PONE-D-21-19666**

**Manuscript Title:** Heart rate variability before and after a race season in Thoroughbred horses and

Standardbred trotters with different training experience

**ABSTRACT**

Lines: 12-14: Names of the investigated groups should be changed – please, see the comment below (Mat. and methods).

Line 17: Abstract section is is not the right place to discuss the obtained results (“which may be the result of…”).

Line 20: Another interpretation of the results (“indicating decrease…”). Please present this as a conclusion from the study, if it is important. And please remember that the interpretation of the obtained results should be discussed in Discussion section only. I think the authors fell into a kind of trap because they did not separate the Results and Discussion sections in the main text of the manuscript. Therefore, in the Abstract section, they used a similar type of description that is not appropriate in this place.

Line 23 and 26: The next comment concerns the marking of statistical significance. It is generally recognized that if there is a significance of differences in results, we consider them as a P≤0.05 or P≤0.01. In my opinion, they are more readable than those in the text (e.g. P=0.030 or P=0.012).

Line 27: Another interpretation of the results.

Lines 31-32: Another interpretation of the results.

Line 32: This statement cannot be considered a research conclusion (please note that, in its current form it is only the authors' assumption).

It seems that in the Abstract there is too little information about the material on which the experiment was conducted and about the methods used. Hence, there are disproportionately many results presented and their interpretations (sic!). Lastly, there are no unequivocal conclusions, only the authors' assumptions (e.g. “The present result suggest...”). Please consider this form: “The present results **indicate** that…”.

**INTRODUCTION**

Line 41: What approaches do the authors mean? Please explain this statement or provide examples from the available literature.

Lines 44-46: First, HR and HRV are two completely separate indicators. Please do not describe them together because it may be mistakenly perceived by the readers. Secondly, if there are examples of HRV parameters, please provide more than one!

Lines 51-61. I cannot agree with the Authors at all. There are many studies on the effects of training on horse HRV parameters, e.g. Frick et al. doi: 10.1111/jvim.15358), Munsters et al. (doi: 10.1017/S1751731112002327), Younes et al. (doi: 10.3389/fphys.2016.00155), Christensen et al. (doi.org/10.1016/j.physbeh.2014.01.024), Kowalik et al. (doi: 10.1111/asj.12671). These studies are not more than 15-20 years old, so please remove this remark entirely from the work. I especially recommend reading the last mentioned work (doi: 10.1111/asj.12671), it contains a well-presented problem of research on HRV in racehorses. In view of the above, I propose to rewrite this whole paragraph, taking into account the latest worldwide research on HRV.

Lines 64-66: Once again, I disagree with the Authors - this method (HRV assessment) has been used for years and cannot be considered as innovative in equine sport medicine. Please carefully consider modifying the purpose of the research performed.

**MATERIAL AND METHODS**

Lines 71-72: Which institution approved the research? Please add the detailed name and seat of the University (city, country etc.).

Lines 74-76: In my opinion, the descriptions of the studied groups of horses are debatable. The Authors do not provide the real/actual age of the tested horses, but only describe their training experience, namely as young horses they consider horses without training experience, and as adults - horses with 1-3 years of experience. This is unacceptable even from a physiological point of view, since both horses (“young” and “adult”) can be adult in fact. Therefore, firstly, information about the real age of the tested horses in the groups should be added (in the text or as separate table), and secondly - I recommend changing the names of the groups to, for example, "untrained" and "trained".

Line 80: What was the potential reason for getting "poor quality HRV recordings"? Why were horses with this writing disorder completely excluded from experience? This statement suggests an error in the measuring apparatus, was it not possible to correct its settings?

Line 83: “after 12 weeks of training” What kind of training? Please refer to the legend to Figure 1 and standardize the type of training used in horses (was it standard training? If so - please write about it here).

Lines 90-93: It is somewhat incomprehensible that the horses identified as YOUNG had exactly the same training program as ADULT. This may suggest that, being untrained, they will have problems with keeping up with the horse's performance. Or vice versa, the ADULT horses did not have loads consistent with their high degree of training advancement. In such a situation, the division into these groups of horses is pointless. Please, explain it more precisely, especially since some training loads differences have been described above.

Paragraph 95-105: There is a lot of questions here. Since pre-training / seasonal recordings started in April, and post-training recordings in August, why do the Authors stated that the study lasted 12 weeks? In fact, from the last week of April to the first week of August there are 14 weeks. So, please precise it. My second question is why the Authors used the term “post-seasonal” recordings here? Is the training season really over in August? If not, maybe it would be better to use the terms "pre-training recordings and" post-training recordings "? Please, consider it.

Line 97: “…intensive training period.” – as mentioned above, after a careful analysis of the training schedule, doubts arise as to its really high intensity. On the basis of what parameters (e.g. LA blood level) the Authors assessed the training intensity in both groups of horses? Such information **is necessary** to confirm whether the training used was really intense. Please provide such data, if possible. If not, please do not use the phrase "intensive training period".

Next question concern the timing of IBI recording. Namely, the authors first note that for such tests, horses should rest without any normal morning activities (see lines: 100 & 101), and then describe that the measurement of IBI was taken after all these activities. Do these statements sound contradictory? In fact, the Authors later mention that the horses had 2 hours of rest, but did this time allow the animals to return to their emotional resting state? How was this confirmed?

Finally, the last comment on this paragraph concerns the term used in line 106. Namely, it is about "development" - in the context of the whole sentence it suggest that at the beginning of the study, some of the horses were undeveloped (somatically? too young? others reasons?). Maybe it would be better to change the word to “experience”, for example?

Lines 117-119: Please do not discuss your chosen method (HF) in this chapter! If in doubt, please discuss it in the Discussion section.

Line 123: why “in farm animals”? or only in farm animals? Can you please expand on this statement?

Line 126: “young vs. adult” – please read my earlier comment.

Line 127: “pre-season vs. post-season” – please consider to change the name the factors / groups to “pre-training” and “post-training”. I suggest this change because in different countries the training season covers usually a longer period (for example from March to October), depending on national regulations.

Lines 133-134: The description of SWC parameter is not necessary here – it should be given in Material and Methods, HRV analysis sub-section.

Line 136: as was mentioned before: not “for adult and young horses”, but for example “trained and untrained horses” .

Line 140: Please, give the full name of the statistical software, with the manufacturer name, and country of origin.

**RESULTS AND DISCUSSION**

First of all, in accordance to PLOS One Authors’ guidance, the “middle section” of the manuscript should consist of following sections: Materials and Methods, Results, Discussion, and Conclusions (optional). So please consider splitting the current Results and Discussion section. This will facilitate a substantive discussion of the obtained results and drawing appropriate conclusions. In the current version, the lack of such a division introduces some chaos and causes that the obtained results are practically unnoticeable.

Moreover, the combination of these subsections in one probably also meant that the authors did not provide any significant conclusions from the study. Namely, in the Conclusion section, the Authors only reported that 12 weeks of training changed the ANS status of the tested animals. The remaining content of this section seems to be the Authors' deliberations only.

Tables and Figures are clear.

References: All of 55 references are cited in the text, and all are given in accordance with the guidelines for the Authors.

Finally, I do not recommend this manuscript in current version. I hope that my comments will help to improve the text of the manuscript and it will be finally published in PLOS ONE.
